# Insights into the Structures of Bilirubin and Biliverdin from Vibrational and Electronic Circular Dichroism: History and Perspectives

**DOI:** 10.3390/molecules28062564

**Published:** 2023-03-11

**Authors:** Giovanna Longhi, Simone Ghidinelli, Sergio Abbate, Giuseppe Mazzeo, Marco Fusè, Stefan E. Boiadjiev, David A. Lightner

**Affiliations:** 1 Dipartimento di Medicina Molecolare e Traslazionale, Università di Brescia, Viale Europa 11, 25123 Brescia, Italy; 2 Istituto Nazionale di Ottica (INO) CNR, Research Unit of Brescia, c/o CSMT via Branze 45, 25123 Brescia, Italy; 3 Regional Health Inspectorate, 7 Prince Al. Battenberg I Str., 5800 Pleven, Bulgaria; 4 Chemistry Department, University of Nevada, Reno, NV 89557, USA

**Keywords:** bilirubin, biliverdin, structural and configurational aspects, circular dichroism, deracemization, anesthetics

## Abstract

In this work we review research activities on a few of the most relevant structural aspects of bilirubin (BR) and biliverdin (BV). Special attention is paid to the exocyclic C=C bonds being in mostly *Z* rather than *E* configurations, and to the overall conformation being essentially different for BR and BV due to the presence or absence of the double C=C bond at C-10. In both cases, racemic mixtures of each compound of either ***M*** or ***P*** configuration are present in achiral solutions; however, imbalance between the two configurations may be easily achieved. In particular, results based on chiroptical spectroscopies, both electronic and vibrational circular dichroism (ECD and VCD) methods, are presented for chirally derivatized BR and BV molecules. Finally, we review deracemization experiments monitored with ECD data from our lab for BR in the presence of serum albumin and anesthetic compounds.

## 1. Introduction and Historical Note

In 1989, Heinz Falk wrote in his indispensable book: “Although bile pigment chemistry originated in the early years of this century, detailed information on the structural aspects of these compounds became available only with the advent of modern spectroscopic methods”. Here we review some results from chiroptical spectroscopic methods, particularly electronic circular dichroism (ECD) and vibrational circular dichroism (VCD), in the investigation of bile pigment compounds, especially bilirubin (BR), biliverdin (BV) and related molecules. Indeed, still following Falk [1] and another book from one of the authors of this review [2], chiroptical spectroscopies are found to be particularly invaluable, since “the chemistry of linear oligopyrroles provides a wealth of stereochemical isomerism”.

We will also report some new experiments in addition to those already present in the literature to show how the exquisite sensitivity of these molecules to chiral environments make them ideal candidates as biologically compatible chiroptical probes.

### 1.1. Fundamentals and Basic Chemistry: Foundations

**Figure 1:**
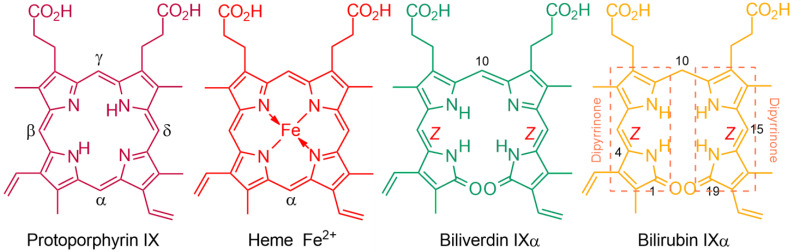
Chemical structures of the pigments of protoporphyrin IX, blood (heme), biliverdin IXα, and bilirubin IXα, the bile pigments that arise from its catabolism in mammals. The stereochemistry of the exocyclic carbon–carbon double bonds follows from heme and was proven incontrovertibly.

Molecules, the compounds’ fundamental explications, are demarcated and, to some extent, defined by their molecular formulas and structures: constitutional, configurational and conformational. The molecular formulas of the tetrapyrrole pigments of bile, biliverdin (C_33_H_34_N_4_O_6_) and bilirubin (C_33_H_36_N_4_O_6_) (Figure 1), were only arduously revealed in the early days of organic chemistry after a long struggle to isolate pure compounds and determine their molecular weights [1,2]. Yet, the latter were not firmly established until the pigments were synthesized. The monumental constitutional structure elucidations by total syntheses of pyrroles and their pigments from the group of Hans Fischer at the Technische Universität-Muenchen (TUM) during 1920–1945 [3,4,5] made it possible for a cascade of future investigations by designed chemical transformations, photochemistry, spectroscopy, calculations and in medicine [1,2].

After a decade-long effort, in a tour de force of 1920s-era organic chemical total synthesis, Fischer and Zeile [6] proved the structure of protoporphyrin-IX (C_34_H_34_N_4_O_4_), the red tetrapyrrole pigment of blood and prosthetic group of iron-containing heme, by total synthesis. For this, Fischer was awarded the Nobel Prize in Chemistry in 1930. Reports of the subsequent total syntheses of the green (biliverdin) and yellow (bilirubin) bile pigments produced by catabolism of heme were published during the middle of World War 2 [2,7]. Were it not for that war, and the associated destruction of his labs, Fischer might have won a second Nobel Prize for his pioneering bile pigment syntheses and his emerging synthesis of the tetrapyrrole plant pigment, chlorophyll.

**Figure 2:**
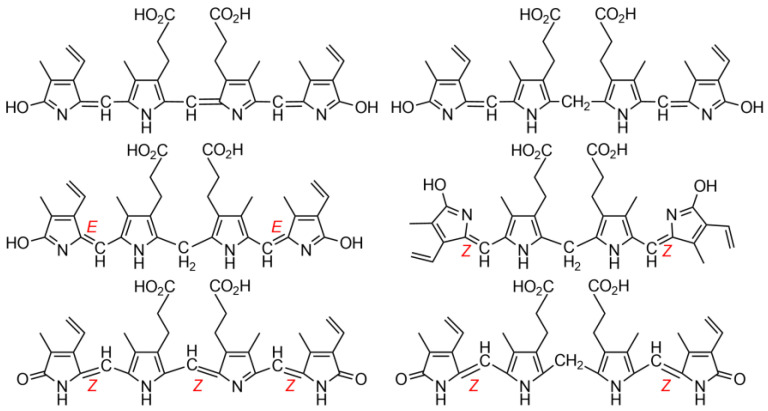
Line drawing graphical representations of BV (**top left**) and BR (**top right**) according to Hans Fischer. (**Middle**) E,E and Z,Z structures of BR according to Lemberg [8]. (**Bottom**) all Z correct linear structures for BV (**left**) and BR (**right**).

Fischer’s constitutional structures of the bile pigments, defined as tetrapyrrole dicarboxylic acids, were complete in most aspects, but lacked: (*i*) clarity at the end rings, for which Fischer preferred lactims; (*ii*) and most importantly, designation of stereochemistry at their exocyclic carbon–carbon double bonds that were neither *cis* nor *trans* (*Z* nor *E*), (Figure 2) [2].

(*i*) Fischer preferred lactims because he believed that dipyrrinones formed acetate esters by reacting with acetyl chloride. Nevertheless, he did recognize that possible lactam tautomers could not then be distinguished in the laboratory. Comments will follow below in discussing the more recent findings on this topic. (*ii*) It is improbable that Fischer did not know of the possibility of configurational stereochemistry about the exocyclic C=C double bonds and its possible influence on molecular properties. The then well-known configurationally isomeric 1,4-butenedioic acids, maleic and fumaric, had been known since the 1870s [9,10] and were included in Fisher’s elementary organic chemistry student lectures at the TUM [11]. In such lectures, the stereochemistry of the exocyclic double bonds of BR and BV were similarly undesignated [4,5,12]. This, despite the fact that the macrocyclic structure of their biological precursor, heme, requires the exocyclic C=C bonds connecting the four pyrrole rings to be in the *Z*-configuration. Fischer, ever the exact experimentalist, apparently could not allow himself to designate a stereochemistry that he could not yet prove but only speculate about.

Why should something as simple and fundamental as C=C stereochemistry be important?

**Figure 3:**
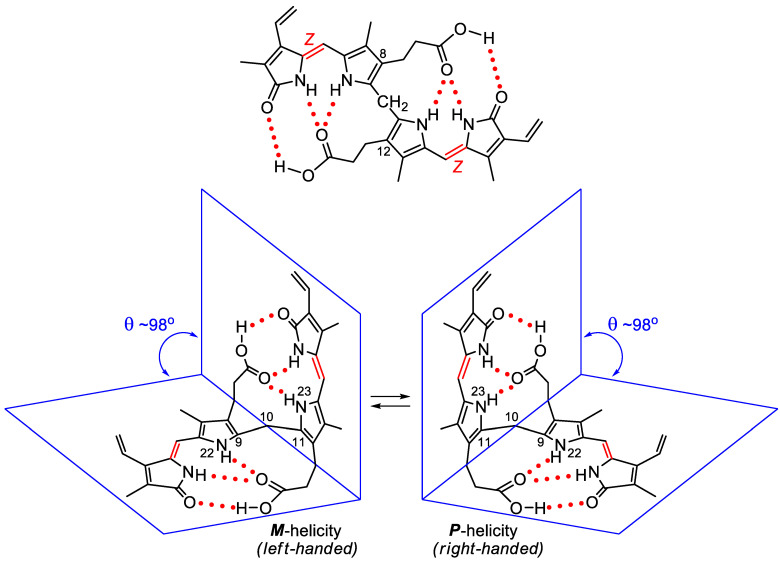
(**Upper**) Intramolecularly hydrogen-bonded bilirubin structure. (**Lower**) Most stable, interconverting conformations of intramolecularly hydrogen-bonded bilirubin.

This is answered by the differing properties of a classic example. In the configurationally isomeric 4-carbon unsaturated dicarboxylic acids, maleic and fumaric acids (HOOC-CH=CH-COOH), the *cis* (*Z*) diacid, maleic, melts at 130 °C, whereas *trans* (*E*), fumaric, melts at 287 °C. Maleic is more soluble in water (50 g/100 mL), whereas fumaric is less soluble (0.6 g/100 mL). Maleic acid forms a cyclic anhydride upon mild heating; fumaric acid first requires a high energy C=C isomerization to maleic. Similarly, the dipyrrinone components of BR may exist in *Z* or *E* configurations about the exocyclic C=C bonds, with the *E* being sterically more crowded and less stable than the *Z* [1]. As with the maleic and fumaric acids, one might expect to see differences in m.p. and solubility in configurationally isomeric bilirubins. In fact, the *E*-isomers can be isolated but are far less stable. The most stable configuration of BR has been shown to be (4*Z*, 15*Z*) [1,2], which is further stabilized by intramolecular hydrogen bonding between the propionic acids and their opposing dipyrrinones (Figure 3, further below) [13,14,15]. As with the dipyrrinones themselves, these components of BR can be converted from *Z* to *E* by visible/blue light, but the *Z* isomers are the more stable, and the *E* isomers of BR convert back to *Z* under the influence of light or thermally. Yet all of the configurational isomers can be separated by HPLC, with the *E* isomers exhibiting greater polarity and the ability to slip across the liver into bile without glucuronidation [16]. This is one of the bases for success in treating the jaundiced neonate with light (phototherapy) [17].

Why then did the incorrect *E*-isomer graphics gain such prominence in the literature?

Shortly after Fischer’s death on 31 March 1945, as WW2 in Europe was sputtering to its end, one of his contemporaries, Lemberg, explained in his highly respected book with Legge [8], and earlier in lectures, that in the absence of contradictory evidence and without direct proof, it would seem logical that the pigments derived from heme should carry forth the same exocyclic C=C configuration that were present in the heme macrocycle [2]. Thus, he deduced that the exocyclic C=C bonds of BR and BV would all have the *Z*-configuration (Figure 1). He noted, however, that his *E*-configuration graphical representations would fit more compactly than his *Z* into linear drawings of the pigments. And so it was noted that, even in recent times, the unproven, scientifically illogical *E*-configurations dominate wherever the exocyclic C=C bonds were expressed in linear representations of the pigments [18,19]. Surprisingly, even after the *Z*-stereochemistry of the pigments in solution became firmly established by spectroscopy [1,2], the *E* continued to be designated without Lemberg’s qualification and propagated in published work [20,21,22,23]. Yet, experimental assignment of the exocyclic carbon–carbon double bonds of BR is of absolute importance to understanding its physico-chemical properties and metabolism [24,25].

### 1.2. Experimental and Computational Structure Determinations

Some 45 years after the seminal papers and books of Fischer [3,4,5,6,7] and the publication of the book by Lemberg and Legge [8], with the advent of modern spectroscopy, X-ray spectroscopy settled the final details of the bile pigment constitutional structure and removed all uncertainties of configuration. It proved that the pyrrole end rings adopted the lactam (NH-C=O) and not the lactim (hydroxypyrrole, N=C-OH) tautomer. Fischer preferred the lactim, which was expressed by others in various publications, books and lectures, including medical, for decades following. However, the X-ray crystallographic studies of verdins by Sheldrick [26] and BR by Bonnett [27,28] and others [29,30,31,32] confirmed the lactam structure, with both pigments showing the characteristic shorter amide C=O bond lengths. The lactam tautomer was doubly confirmed by Falk from X-ray photoelectron spectroscopy, with a focus on the end-ring nitrogens [33] and by ^15^N-NMR for the pigments in solution [34].

While the *Z*-configurations of the exocyclic carbon–carbon double bonds at C-4, C-10 and C-15 of the verdin, and C-4 and C-15 of the rubin were found in the solid, they also were proved for the pigments in solution [13] by ^1^H-NMR nuclear Overhauser effect (NOE) studies. Thus, NOE enhancements were detected between the =CH hydrogens at C-5, C-10 and C-15 and the nearest neighboring CH_3_ or CH_2_ groups of the flanking pyrrole beta substituents of verdins [1,2,35,36,37] and bilirubin [1,2,38,39,40]. Nonetheless, the unstable *E*-configurations are occasionally found represented even now in the literature of bile pigments [20].

**Figure 4:**
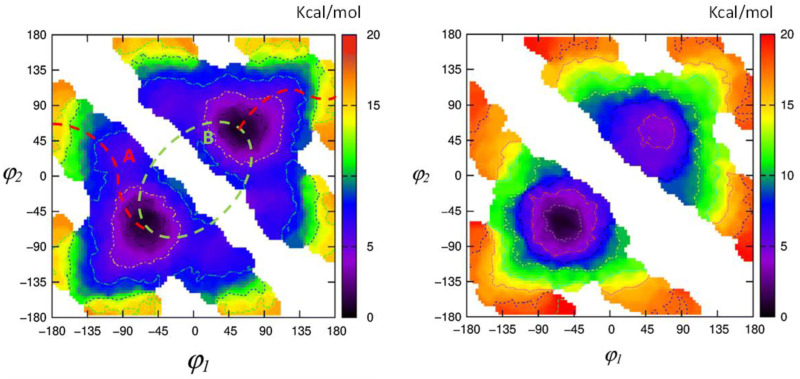
(**Left panel**) Free-energy surface obtained by metadynamics simulation of bilirubin in chloroform solution. Energy values are in kcal mol^−1^; axes refer to the dihedral angles φ_1_ and φ_2_ pivoting on C-10. The two lowest-energy routes (A and B) connecting the ***M*** and ***P*** conformational enantiomers are also reported. Along path A (red), the interconversion barrier is about 16.3 kcal mol^−1^; along path B (light green), the energy barrier is higher than 20 kcal/mol. (**Right panel**) Free-energy surface obtained by metadynamics simulation of chiral (βS,β′S)-Dimethylmesobilirubin-XIIIα in chloroform solution. The minimum corresponding to the ***M*** configuration is at a lower energy (by a value of 4.1 kcal mol^−^1) than the one for the ***P*** configuration. (Adapted from [45]).

It is relevant to state that X-ray crystallography also revealed important aspects of the conformation of BV and BR. In the solid, the former adopts a porphyrin-like shape that is not planar but twisted into a helical lockwasher shape with a 16° dihedral angle pivoting on C-10 [33,37]. Thus, BV is intrinsically chiral, but the two enantiomeric helices, which easily interconvert, are equally present in isotropic solutions of BV and, consequently, exhibit no optical activity. Yet, when the pigment is constrained, Falk showed that bridging the lactam nitrogens by a -CH_2_- unit leads to a verdin that can be resolved into enantiomers, with an interconversion activation barrier of 105 kJ/mol at 333 K [41]. Similarly, X-ray studies showed that BR adopts a ridge-tile shape, with the two dipyrrinones rotated 60° about C-C bonds at C-10 so as to exhibit a ridge-tile shape in which two planar dipyrrinones form a dihedral angle of about 100 deg and are connected to the opposing propionic acids by intramolecular hydrogen bonds [1,27,28,29,30,31,32]. Two rapidly interconverting enantiomeric conformations prevail in solution with an activation barrier (Figure 3) of ΔG^‡^ 74.9 kJ/mol at 53 °C and an interconversion rate of 7.2 ± 0.4 s^−1^ [42] or 3–95 s^−1^ at 50–95 °C, according to [43]. Recent metadynamics studies on BR, in accord with earlier MM–Sybyl studies [44,45], allow one to graphically follow the interconversion paths between different conformers bearing chiral character and to evaluate the energetics thereof (see Figure 4).

### 1.3. Spectroscopic Studies

Knowledge of the bile pigments’ secondary structures so essential to understanding the pigment metabolism and biology and spectroscopic behavior remained incompletely understood until more recently. Investigations of the conformational structures of the pigments have revealed a multiplicity of pigment conformations, with the most stable having been tested out by 2D-NMR [43], electronic [1,2,44] and vibrational circular dichroism, etc. [46,47,48,49,50,51,52] These characteristics and spectroscopic properties have come into play in solutions of biliverdin-derived photonic materials for photoimaging and phototherapy and bilirubin-derived materials for photoimaging and phototherapy [53], cancer diagnosis photoacoustic imaging, phototherapy and anti-inflammation therapy [54] and BV spectroscopy with an eye toward understanding phytochrome and photoactivity [55,56].

With the constitutional and configurational structure of BR now firmly established, some conformational peculiarities are worth considering. As noted above, BR is not planar but bent about the central CH_2_ unit. Its two dipyrrinone components can rotate like paddles about the central CH_2_ so as to bring them into sufficiently close proximity to the propionic acid CO_2_H groups to allow them to engage in intramolecular hydrogen bonding (Figure 3). Not only is such intramolecular hydrogen bonding a conformation stabilizing factor but it is one that considerably alters the intrinsic solubility of the pigment [2]. A most intriguing aspect of the 3D structure of intramolecularly hydrogen-bonded BR is its ability to fold into either of two interconverting enantiomeric conformations (Figure 3) [44]. It is this conformation of BR that has attracted considerable attention and that has been studied spectroscopically and by theoretical calculations in recent years [2,45].

## 2. Chiroptical Spectroscopies

### 2.1. The Advent of Bilirubinoid Chiroptical Spectroscopies

In the following, we recall how circular dichroism in its two main forms—electronic in the UV-vis range (ECD) and vibrational in the IR range (VCD)—came into play and helped to clarify some of the configurational/conformational aspects discussed above, which are so important in the biomedical functioning of BR and BV. Since neither BV nor BR is chiral, the first important contribution came from the ECD studies of Moscowitz and collaborators on urobilin and stercobilin-HCl [57,58,59], working on naturally optically active samples previously isolated by Watson [60,61]. Moscowitz was able to recognize in the ECD data for the two natural compounds the motif of inherently dissymmetric chromophore, resulting in a monosignate spectrum, which was recognized later in the ECD spectra of BV as well as of hexahelicene [62,63].

Despite the fact that BR and BV are both achiral, optical activity may be induced by the chiral environment: in 1966, induced optical rotation was observed by Scholtan and Gloxhuber [64] while characterizing BR albumin binding. In 1972, Blauer et al. [65] recorded ECD spectra of BR in serum albumin aqueous solutions: they observed bisignated couplets and, while hypothesizing different possible conformers, interpreted the bisignated feature as coupling between electric dipole transition moments of bound BR leading to exciton splitting.

### 2.2. Chiral Derivatives (ECD and VCD Experiments and Calculations)

The observation of an ECD signal, and thus the possibility to monitor different conformations in solution, led the Lightner group to synthesize a large variety of chiral derivatives of BR in order to achieve controlled imbalance between ***M*** and ***P*** helical enantiomeric structures, depending on the nature and position of the substituent. In particular, chemical modifications of propionic acid chains induce modulation of the ECD spectrum, thus giving an important indication about the role of H-bonding on the molecular conformation in solution while also testing different solvents.

**Figure 5:**
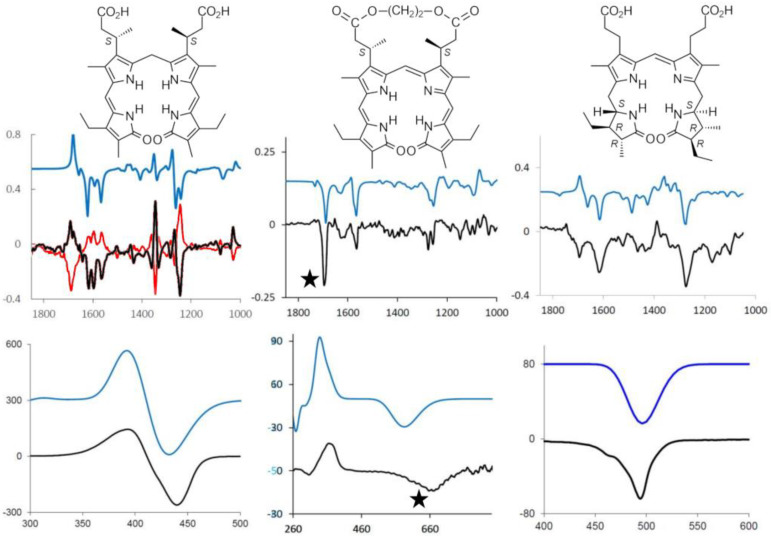
VCD (**top**) and ECD (**bottom**) of “chiral” BR (β*R*,β′*R*)– and (β*S*,β′*S*)–dimethylmesobilirubin–XIIIα (only the latter structure is shown) in red and black, respectively: (**left**), “chiral” BV (β*S*, β’*S*)–dimethylmesobiliverdin–XIIIα; cyclic ester (**center**) and *l*–stercobilin hydrochloride (**right**): experimental spectra are reported in black and calculated spectra in blue. See refs. [48,51,52], respectively, for details of calculations on the three molecules. For the chiral BV, the characteristic bands for ***M***/***P*** helicity are starred (***M*** in this case)(*vide infra)*.

These observations, recently supported also by VCD and quantum mechanical calculations rooted in density functional theory (DFT), confirm the persistence of the 6-H bonded structure in solution; however, the propensity to form a ridge-tile structure is maintained also when the six H-bonds are not possible due to chemical modifications [66,67]. This is confirmed by geometry DFT optimization and by the good correspondence between theoretical and experimental spectra (see Figure 5, as an example) [48].

Analogous chemical substitutions have been considered for BV, introducing also, in this case, chiral centers in order to exploit chiroptical spectroscopies and ancillary calculations to test the importance of propionic acid chains by variously modifying them. An illustration of the comparison of experimental and calculated spectra, both ECD and VCD, is given for a chiral BR derivative, for a chiral BV derivative and for *l*-stercobilin hydrochloride in Figure 5. In passing, we may notice that not only ECD spectra for BR-chiral derivatives exhibit bisignate features, differently from the monosignated ECD spectra of BV-chiral derivatives and of *l*-stercobilin, but VCD in the mid-IR also behave similarly. Indeed, BV-chiral derivatives and *l*-stercobilin exhibit almost monosignated negative VCD spectra in the mid-IR range, whereas the VCD spectra of BR chiral derivatives are rich in bisignate features. This reflects the two 3D-structural motifs, namely lockwasher and ridge-tile, typical of BV and BR, respectively.

To the best of our knowledge, the first group that used VCD spectroscopy aside from the most common ECD technique to study BR and BV was Urbanová, who examined the achiral compounds BR and BV in different chiral environments [46,47,49]. In general, the advantage of VCD spectroscopy is the presence of many bands, a characteristic possibly providing a stringent check of the computational model based on the normal mode analysis within the DFT frame. 

In addition, the technique is quite sensitive to subtle conformational details and solvent interactions. In the case of organic aprotic solvents such as chloroform or carbon tetrachloride, a PCM (Polarizable Continuum Model) model [68] is often sufficient to obtain good calculation–experiment correspondence. Other solvents (or the presence of counterions) may instead have a large influence on the conformational landscape, in which case an MD (Molecular Dynamics) simulation could be necessary in order to include explicit solvent molecules with subsequent better spectra calculations based on an adequate number of snapshots.

**Figure 6:**
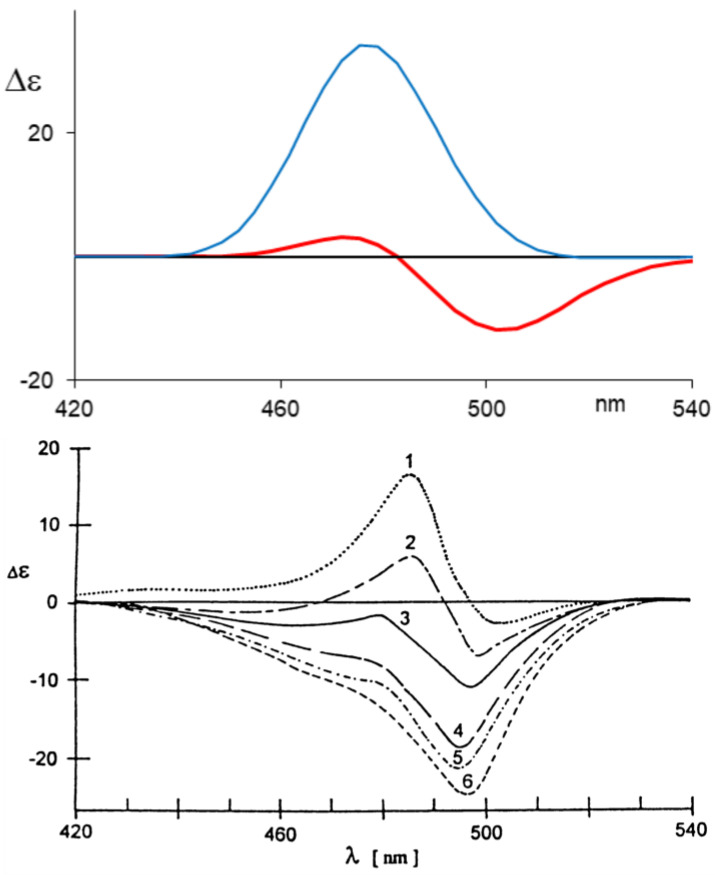
(**Top**) Calculated ECD spectra for the least populated conformer (blue) and for the Boltzmann-weighed enantiomeric conformers at T = 160 K. (**Bottom**) Experimental ECD spectra of *l*-stercobilin in ethanol–glycerol solution (9:1 *v*/*v*) at variable temperatures from T = 163 K (1) to T = 297 K (6), (revised from ref. [59]).

For example, we have considered *l*-stercobilin-HCl in both CD_2_Cl_2_ for VCD [52] and in methanol for ECD, inspired by the experimental results by Lightner and Moscowitz [59]. In both cases, a preliminary MD treatment to obtain dynamically allowed geometries gives better results than just implicit solvent modelling. A notable example is represented by ECD in methanol (more precisely, the experiment was conducted in methanol:glycerol 9:1 *v*/*v*) at different temperatures. The monosignate negative CD band recorded in chloroform for *l*-stercobilin-HCl is observed also at room temperature in methanol:glycerol 9:1 solution, while lowering the temperature caused the differently populated structures obtained also by simulation to exhibit positive (and higher energy) features (Figure 6). This proves that the two enantiomeric lockwasher-type conformers are present to differing amounts in solution and, by lowering the temperature, the relative weight between the thermodynamically less-favored and more-favored conformer changes.

### 2.3. Aggregation and Sensing (of Bilirubin and Biliverdin Chiral Derivatives)

When considering spectroscopic features in detail, one needs become aware of the influence of aggregation effects: in this instance, taking into account not only intramolecular but also intermolecular interactions may be crucial to interpreting experimental spectra correctly.

Vapor pressure osmometry and ultracentrifugation experiments [69,70] have evidenced the presence of BR-derivative aggregates, also suggesting a dimeric structure. Considering VCD measurements, which are usually performed at high concentration to produce reliable signals, it is mandatory to check for possible aggregation phenomena; in fact, tests conducted at different concentrations of both running VCD and ECD measurements confirmed the presence of aggregates. The proposed dimeric structure for BR-chiral derivatives appears plausible and accounts for the spectroscopic changes observed upon increasing concentration since DFT calculations show good correspondence with the observed features [50].

Even more interesting is the behavior observed for BV and its chiral derivatives, showing high sensitivity to both pH changes [1] and to the presence of metal cations [1], as evidenced also by visible absorption spectra. The use of chiral derivatives with a delicate equilibrium between ***M*** and ***P*** forms shows that the imbalance toward one chiral form or the other may be highly influenced by pH, thus justifying the use of these molecules as molecular sensors [51]. In this instance, the set of derivatives in which the original propionic acids are belted together is a good example: On one hand, it was observed [71] how the length of the CH_2_ unit belt (see Figure 5, center) gives rise to either ***M*** or ***P*** prevalence on the same (*S,S*) series as detected by ECD and VCD; the conformational unbalance is also well accounted for by DFT calculations. On the other hand, such labile chiral induction is susceptible to change under environment perturbations such as pH variations. The monosignate characteristics of the first CD feature in the visible range correlates with the dominant VCD features at 1700 cm^−1^ (see Figure 5 center, features indicated with stars).

**Figure 7:**
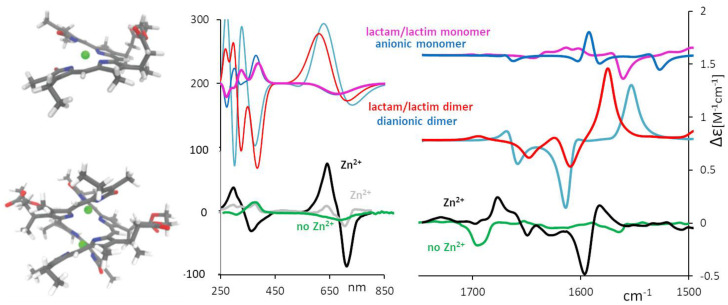
(**Middle** and **right**) Calculated ECD and VCD spectra for chiral BV derivative (see Figure 5) are shown for a dianionic dimer (blue curve), lactam/lactim dimer (red curve), weighted average of anionic monomer (blue curve) and weighted average of lactam/lactim monomer (pink curve). They are compared to the experimental ECD and VCD spectra recorded in methanol and chloroform solutions, respectively: green curve, no added salt; black curve, concentrated 5 × 10^−3^ M solution of Zn-complex; gray curve, diluted solution 2 × 10^−6^ M of Zn-complex. (**Left**) Corresponding monomer and dimer models optimized to calculate spectra.

It is interesting to point out that, while in the absence of metal ions, mostly monosignate VCD features are present, strong bisignate VCD features appear at about 1600 cm^−1^ in the presence of metal ions, −/+ in order of decreasing wavenumber. Such features can be assigned to species of the chiral BV-derivative present at high concentrations s, as needed for running VCD spectra. A systematic study was conducted in ref. [51], where an aggregation equilibrium constant was also derived. In Figure 7, we show the experimental VCD spectra of the chiral BV of Figure 5 in the presence of Zn^2+^. DFT calculations for the monomer species reported in the same Figure are unable to represent the doublet at 1600 cm^−1^, which is instead justified by DFT calculations for a dimer model. For ECD data in presence of Zn^2+^ at various concentrations, one is also able to observe that an intense doublet indicative of a dimeric structure appears at concentration values comparable to those used for VCD. We remark that the dimer formation had been hypothesized and observed with X-ray diffraction on different BV-derivatives by Furhop et al. [72], as well as by Bonfiglio et al. [73], in close agreement to what is shown in Figure 7.

## 3. Deracemization with Chiral Agents

Several deracemization effects on BR and BV have been observed in various chiral environments. 

### 3.1. Small Molecules

Induced circular dichroism on natural BR and analogs has been observed in association with (relatively small) chiral agents. Examples are given by BR in dichloromethane solution with added chiral cinchona alkaloids [74] or even simple, optically active amines [74,75] or by achiral mesobilirubin in the presence of chiral sulfoxides: in both cases, the “unbalancing” of the two enantiomeric ridge-tile conformers promotes the typical exciton coupling-induced CD signal [76,77].

### 3.2. Cyclodextrin and Surfactants

Other supramolecular interactions inducing optical activity are represented by cyclodextrins [78,79], chiral surfactants [80] and micelles [81]. Due to the preferred ridge-tile conformation and to the ease of hydrophobic interactions, BR deracemization can be used as a marker of micellar aggregates’ chirality [82]. This last example is particularly interesting since it concerns chiral recognition of biomembrane models using BR as a probe for detecting the chirality of the aggregates themselves [83]. The observed chiral imbalance was found to depend on concentration and on the hydrophobic alkyl chain length, confirming the role of lipid structure in the membrane–bilirubin interaction. Such sensitivity to the chiral environment suggested the possibility that chirality plays a role in BR neurotoxicity, since the stereodynamics of BR interacting with alkyl chains may perturb membrane dynamics [84].

### 3.3. Serum Albumin: Discussion of an Example Dealing with Pharmaceutically Active Molecules

A separate paragraph is needed to consider BR and BV interactions with human serum albumin (HSA), not only because it was the first system in which optical activity was observed [64], but also because of the biological/medical implications [24], serum albumin being the principal transport protein [81,85,86,87,88]. BR and BV have been considered also in interaction with enzymes such that characterization can also be followed, taking advantage of chiroptical techniques [89,90].

**Figure 8:**
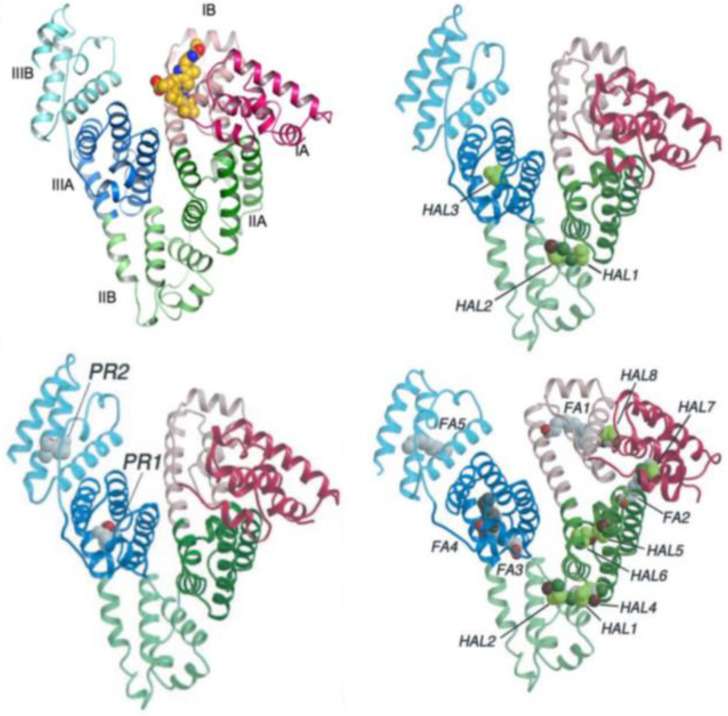
(**Left**, **top**) 3D structure of HSA complexed with 4Z,15E-bilirubin-IXα with the definition of its various domains (see ref. [91]). (**Left**, **bottom**) 3D structure of HSA with the determined binding sites for Propofol (PR1 and PR2). (**Right**, **top**) 3D structure of HSA with the determined binding sites for Halotane at moderate Halotane concentrations. (**Right**, **bottom**) 3D structure of HSA with the determined binding sites for Halotane at high Halotane concentrations (taken with permission from ref. [92]).

Despite the numerous studies regarding the bilirubin–HSA complex, there is not an overall accepted picture at the molecular level. An X-ray structure [91] of a bilirubin–HSA complex indicates site IB (Figure 8) as the domain of the protein complexed with a BR stereoisomer. However, possibly due to the crystallization procedure not having been conducted under safe lights, BR was found to be the 4*Z*,15*E* isomer. The same authors suggest that it is reasonable to assume that the natural 4*Z*,15*Z* isomer interacts with HSA at the same protein site. This is because the same pocket being monitored was found to be occupied by co-crystallized fusidic acid, and fusidic acid is known to competitively displace BR from the protein. 

It is certain that complexation with HSA stabilizes the ***P***-helical form of BR, whereas BSA selects the ***M*** form, as evidenced by the observed CD couplets. Concerning the precise binding site, some debate is present in the literature: ligand-competition experiments suggest the presence of binding sites with different affinity (IIA with highest affinity, followed by IB and IIIA) [93]; other studies tested BR binding to the two separate domains I and II of HSA—in this case, domain I does not give a CD couplet of the correct shape, but domain II, despite giving the correct sign of the CD couplet, also does not show the same intensity (and wavelength) as observed with the whole native HSA [94]. Following the experiments conducted by the Urbanova group [95], it seems that BV binds to the so-called heme site, IB, while for the *Z,Z* form of BR, the principal binding site is IIA. In any case, it is known that allosteric modulation may couple site IIA and the heme site [96,97], which may make interpretation of displacement-competition experiments more difficult. Despite these difficulties, BV and BR have been both used to study drug–HSA binding in competition experiments in order to gain information about pharmaceutically active molecules. 

In the following, we report on the case of anesthetics, including volatile haloethers (Desflurane and Sevoflurane) and an intravenous anesthetic (Propofol). BR, showing spectroscopic activity in the visible region, may be easily applied as a sensitive chiroptical probe of drug–protein interactions.

**Figure 9:**
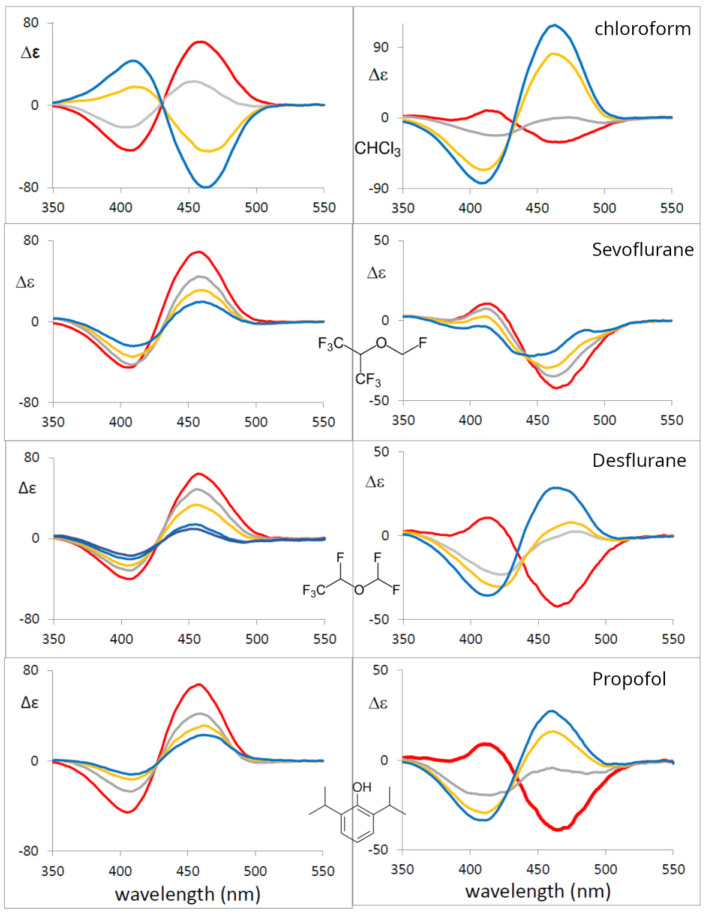
Left: ECD Spectra for HSA + BR + the indicated anesthetics in phosphate buffer solution Right: ECD Spectra for BSA + BR + the indicated anesthetics in phosphate buffer solution (see text). **Color coding**: Red: no anesthetic. Gray, yellow, blue: increasing anesthetic concentrations, in this order (see text).

It was first observed by McDonagh et al. [98] that volatile anesthetics have a remarkable effect on the circular dichroism of BR bound to HSA, causing a Cotton-effect sign inversion upon the addition of halothane, chloroform and diethyl ether. This suggested to us the possibility of also testing other anesthetics in an analogous way. In Figure 9, we present our results on the two proteins, HSA and BSA (bovine serum albumin), complexed with BR, interacting with increasing quantities of chloroform, Sevoflurane, Desflurane and Propofol. (Experiments at BR and serum albumin concentrations of 2.2 mM in 0.1 M phosphate buffer, pH 7.8. Concentrations of anesthetics in HSA: CHCl_3_ (0, 0.025, 0.050, 0.075 M), Sevoflurane (0, 0.04, 0.08, 0.12 M), Desflurane (0, 0.05, 0.1, 0.15, 0.02 M), Propofol (0, 0.015, 0.030, 0.045 M). In BSA: CHCl_3_ (0, 0.015, 0.030, 0.045 M), Sevoflurane (0, 0.025, 0.050, 0.075 M), Desflurane (0, 0.05, 0.1, 0.15, 0.02 M), Propofol (0, 0.015, 0.030, 0.045 M)). It may be observed that the ECD couplet recorded in BSA aqueous solutions shows sign inversion and high intensity in all cases but Sevoflurane; this leads us to conclude that these anesthetics promote a different binding mechanism, inducing and selecting the opposite BR chirality. In the case of HSA, the three tested drugs show weakening but no sign inversion of the ECD couplet, which could be due to either unbinding of BR or, most probably, to the presence of the second form of enantiomeric conformation induced either by interaction with a different pocket of HSA or by a local change in protein conformation. Fluorescence measurements on exactly the same solutions used for CD show intense fluorescence, due to bound BR, at about 532 nm which is nearly constant with anesthetic concentration or shows a slight increase in intensity, thus indicating that the molecule is still bound to the protein (in fact, free BR does not give significant fluorescence [99,100]).

Other studies may be found in the literature concerning the binding process of anesthetic drugs [101,102,103], including considering four-α-helix bundles to model the transmembrane domains [104]; however, it is worthwhile to recall that, in general, crystallization of protein–anesthetic complexes is also not an easy task [105] due to the low solubility of these compounds. In fact, Sevoflurane and Desflurane are rapidly absorbed into the blood circulation system; their solubility in blood is low, therefore a minimal amount needs to be dissolved in blood to be effective, and low solubility correlates with rapidity in inducing anesthesia. Among the possible interaction mechanisms considered between volatile anesthetics and proteins, solvent restructuring, resulting from the release of water weakly bound to anesthetic and anesthetic-binding sites, was surmised to play a major role [101,106]; furthermore, anesthetics may act as a halogen-bond donor [107]. As per Zsila, anesthetics affect protein structure, inducing folding, misfolding, or even aggregation [108].

Considering X-ray data of anesthetics: For HSA complexes, binding sites have been identified for Propofol [92], which binds at two discrete sites on HSA in preformed pockets (Sudlow’s site II, in subdomain IIIA, and subdomain IIIB), and also for Halotane, which binds at three main sites (one in IIIA and two in IIA) (see Figure 8) with high affinity [92]. However, at much higher drug concentrations, additional sites have been identified in this last case. It is worthwhile recalling that competition experiments are frequently used to identify binding sites with the aid of spectroscopic methods [89,109] and isothermal titration calorimetry [110]; recently, high-performance liquid chromatography plus docking modeling has characterized Propofol-binding affinities to various SAs [111].

Considering the results reported in Figure 9, further studies are needed to better identify the BR pocket and the reason for the observed signal changes. Nonetheless, they are a clear indication of strong drug–protein interaction.

## 4. Conclusions

Interaction of BR with proteins is still a topic of great interest [112], both because of BR toxic effects but also because a small increase in BR may have antioxidant and anticancer potentialities. Since BR possesses multiple biological actions [113], the study of BR effects on protein structure and function is still pursued; BR shows, for example, an antioxidant effect on fibrinogen, preventing carbonylation and aggregation and eventually modulating homeostasis [114], while BR and BV have regulatory functions by binding to the peroxisome proliferator-activated receptor-α [115,116].

From the point of view of BR biological implications, particularly for neonatal jaundice phototherapy, from the 1970s, BR photoisomerization has been extensively studied by McDonagh and Lightner [117,118]. Characterization of photoisomers of BR bound to serum albumin is also important [119,120], and the dynamics of photoinduced configurational and structural isomerization are still worth investigating with modern spectroscopic techniques [121] since all details of such processes are not yet completely clear.

These studies have opened the way to discover the possibility of photoimaging and phototherapy applications, based on both BR and BV, which have shown high po-tentialities [54]: among others, great interest has grown about a fluorescent protein, UnaG, from Japanese eel, whose fluorescence is triggered by BR [122], for biliru-bin-decorated nanoparticles with therapeutic applications [123], for BR nanoparticles applied to imaging [124], etc.

All these applications show how BR and BV are still of great relevance and how useful the study of induced CD is to monitor the chiral environment of BR/BV; the latter technique may suggest further applications of chiroptical spectroscopies to gain knowledge useful to the most recent applications.

## Data Availability

Further information available from the authors upon request.
